# Roles of Salicylate Donors in Enhancement of Productivity and Isotacticity of Ziegler–Natta Catalyzed Propylene Polymerization

**DOI:** 10.3390/polym12040883

**Published:** 2020-04-10

**Authors:** Manussada Ratanasak, Jun-ya Hasegawa, Vudhichai Parasuk

**Affiliations:** 1 Institute for Catalysis, Hokkaido University, Kita 21, Nishi 10, Sapporo, Hokkaido 001-0021, Japan; manussada@cat.hokudai.ac.jp (M.R.); hasegawa@cat.hokudai.ac.jp (J.-y.H.); 2 Center of Excellence in Computational Chemistry, Department of Chemistry, Faculty of Science, Chulalongkorn University, Bangkok 10330, Thailand

**Keywords:** salicylate donor, Ziegler–Natta, propylene polymerization, stereoselectivity

## Abstract

Roles of internal salicylate donors (SID) in enhancing activity and stereoselectivity of Ziegler–Natta catalyzed propylene (PP) polymerization were examined using DFT calculations. Five salicylate donors were studied. The chelate mode is the preferred adsorption mode. The linear relationship (R^2^ = 0.96) between calculated adsorption energies (E_ads_) of five SIDs and the experimental PP activities was observed. Thus, the SID with the strongest adsorption energy will provide the highest activity in agreement with our previous studies. Compared with diisobutyl phthalate (DIBP), which is the industrial electron donor, SID has stronger E_ads_. The insertion step, which involves the π-complex formation (∆E_π_) and the insertion activation or intrinsic activation energy (E_a_) for PP polymerization was also examined. The relation between ln(activity) and apparent activation energy (E_a_(app)), which is ∆E_π_ + E_a_ for the primary(1,2)-*re* insertion with R^2^ = 0.99, was observed. The salicylate donor also has a lower E_a_(app) than that of DIBP. This explains the better catalytic performance of SID. Our results also demonstrated that the size and the type of hydrocarbon substituents play a key role in controlling stereoselectivity and activity. In addition, we found a good relationship between E_ads_ and both intrinsic (E_a_) and apparent (E_a_(app)) activation energies of five salicylate donors with R^2^ of 0.90 and 0.97, respectively.

## 1. Introduction

Transition metal-based catalysts for olefin polymerization play a crucial role in the development of novel versatile and environmentally-friendly polymeric materials, including both commodity and specialty polymers [1]. Polypropylene (PP) is among the highest in demand polymer/plastic due to its high-performance characteristics such as high level of stiffness, high melting point compared to other commercial thermoplastics, good resistance to impacts, low production cost, high heat distortion temperature, transparency, and ability to be recycled, etc. [2,3,4]. Most of the commercial catalysts used for PP manufacture are modified from the original Ziegler–Natta (ZN) system by adding organic Lewis bases or electron donors to enhance productivity and isotacticity [5]. Electron donors, which are added during catalyst preparation, are called internal electron donors and those added during the polymerization process are called external electron donors [6]. Since internal electron donors are mixed with the ZN catalyst during preparation, it strongly impacts the active center of the ZN catalyst and the microstructure of PP. Thus, the internal (electron) donor has become a key component for improving the comprehensive properties of polypropylene [7,8,9]. Many classes of chemical compounds [10,11,12,13,14,15] have been employed as internal donors in the ZN-catalyzed propylene polymerization.

**Figure 1:**
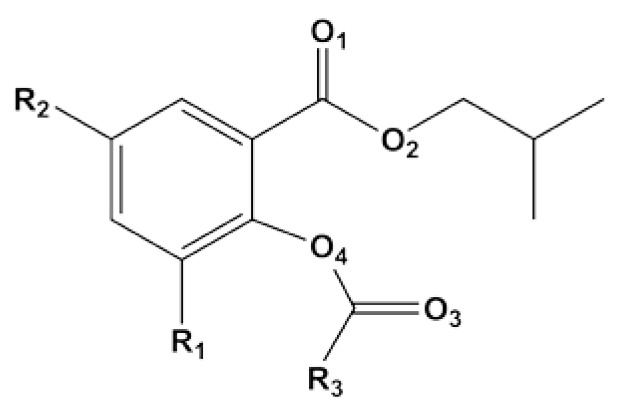
Chemical structure of salicylate donors with different substituent groups at R_1_, R_2_, and R_3_ positions.

Recently, Zhou et al. [16] synthesized five salicylate (SID) compounds with different sizes of hydrocarbon substituents for heterogeneous ZN-catalyzed PP polymerization. They were comprised of isobutyl 2-benzyloxybenzoate (SID-1), isobutyl 2-benzyloxy-3-methyl benzoate (SID-2), isobutyl 2-trimethyloxy-3-methyl benzoate (SID-3), isobutyl 2-benzyloxy-3,5-isopropyl benzoate (SID-4), and isobutyl 2-benzyloxy-3,5-tert-butyl benzoate (SID-5) (see Figure 1, Table 1). These new electron donors are around 2–3 times more effective in enhancing the activity of the ZN catalyst than the industrially used diisobutyl phthalate (DIBP) donor [16].

There have been several theoretical studies on roles of electron donors such as malonates [17], succinates [18,19], 1,3-diethers [19,20,21], phthalates [22,23], methyl benzoates [14,24], ethyl benzoates [25,26], alkoxysilanes [27,28,29], and dibenzoyl sulfide in ZN-catalyzed PP polymerization [23]. Since salicylate compounds are the newest electron donors, no theoretical study on this topic has been reported. The purpose of this work is, therefore, to understand the roles of salicylate donors in enhancing activity and stereoselectivity of the ZN catalyst. Furthermore, to guide the design of future electron donors obtained results were used to compare with our previous theoretical works on other electron donors, especially with DIBP [23].

Many researchers have shown that Ziegler–Natta catalysts are highly regioselective for PP polymerization [17,23,30,31,32]. The accepted mechanism for olefin polymerization was given by Cossee and Arlman [33,34,35] and Brookhart and Green [36]. In principle, the ZN-catalyzed PP polymerization is composed of three steps: insertion, propagation, and termination. The PP insertion is the rate determining step (RDS) [4,37], as well as the stereocontrol step [38]. Thus, the insertion step for ZN-catalyzed PP polymerization with five salicylates as given in Table 1 was investigated. Since the energy of the transition state for the primary (1,2) insertion of propylene to Ti-*i*Bu bond has been reported by several research groups to be lower than that of the secondary (2,1) insertion [17,19,22,23,39]. Therefore, in this work, we considered only the primary (1,2) insertion for the investigation of the roles of salicylate donors in enhancing activity and stereoselectivity of the ZN catalyst.

## 2. Computational Details

### 2.1. Adsorption Modes

The [Mg_13_Cl_26_.Cl_2_Ti] cluster was employed for modeling of the pre-activated MgCl_2_ (110) surface of the ZN catalyst similar to our previous work. [23] The MgCl_2_ (110) surface model has been proven both experimentally and theoretically to be the most stable plane [40,41,42,43,44]. An octahedral shape of a mononuclear Ti atom adsorbing on the MgCl_2_ (110) plane is also a good model for the activated catalyst, and this surface model could reproduce experimental Raman spectra of the MgCl_2_-TiCl_4_ sample [45].

**Figure 2:**
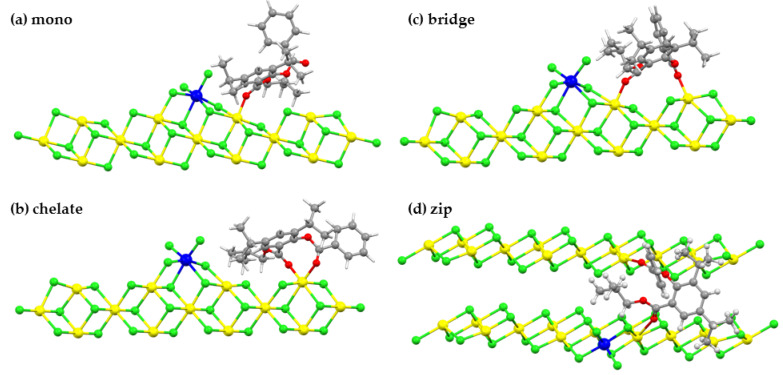
Four different adsorption modes, (**a**) mono, (**b**) chelate, (**c**) bridge, and (**d**) zip of the salicylate donor (SID-4) on the Ziegler–Natta (ZN) catalyst. Color key: Mg, yellow; Ti, blue; Cl, green; O, red; C, gray; H, white.

Four possible adsorption modes, i.e., mono, chelate, bridge, and zip modes, as illustrated in Figure 2, were investigated.

Adsorption energies were obtained using DFT calculations with the B3LYP-D3 functional [46]. This functional has been proven by Cavallo et al. [47] to well reproduce the experimental association energies between TiCl_4_ of fifteen electron donors. The LANL2DZ effective core potentials (ECP) function and basis set were used for Ti atom [48], while the 6-31G(d,p) basis set was used for others. During the optimization of the geometry for the [Mg_13_Cl_26_.Cl_2_Ti] cluster, which represented the MgCl_2_ (110) surface, the four MgCl_2_ units at the top layer, which involved donor and Ti coordination, were relaxed. The remaining MgCl_2_ units were fixed to the MgCl_2_ X-ray structure [49], while the geometry of donor molecules was fully optimized. The adsorption energy (E_ads_) is defined according to Equation (1):

E_ads_ = E(complex) − E(surface) − E(donor) (1)

where E(complex) is the energy of the complex between the electron donor and the TiCl_4_/MgCl_2_ (110) surface. E(surface) and E(donor) are the energies of the TiCl_4_/MgCl_2_ (110) surface and the electron donor, respectively. All DFT calculations were conducted by employing the Gaussian 09, Revision B.01. program [50].

### 2.2. Activation Energies and Stereoselectivities

**Figure 3:**
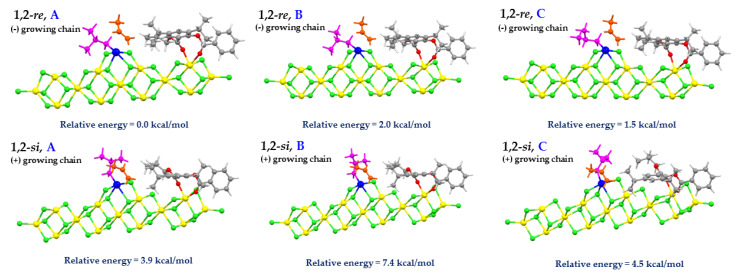
Six possible conformations of the *i*Bu growing chain for the π-complex of 1,2-*re* and 1,2-*si* insertions at the Ti mononuclear on the MgCl_2_ (110) surface in the presence of salicylate donor (SID-4). The insertion of propylene on the front side, 1,2-*re*, A and 1,2-*si*, A. The insertion of propylene on the backside, 1,2-*re*, B and 1,2-*si*, B. The insertion on propylene on the right side, 1,2-*re,* C and 1,2-*si*, C.

The active surface model was constructed by adding the isobutyl (*i*Bu) group to the Ti center of the complex structure between the [Mg_13_Cl_26_.Cl_2_Ti] cluster and the salicylate donor. The use of *i*Bu as the growing chain followed the suggestion of Tom Ziegler et al., [32]. In their article, methyl, propyl, isobutyl, and 2-butyl groups were used as the model for growing polymer chains for investigating the influence of the growing polymer chain on stereoselectivity. Their results revealed that the bulkiness of the polymer chain disfavors propylene insertion, and that the 1,2-*re* insertion is the most favorable. Thus, the stereoselectivity is controlled by the interaction between the propylene and the growing polymer chain. Besides, we have considered (+) and (-) orientations of the *i*Bu growing chain according to Corradini’s model [51,52,53]. Possible orientations of *i*Bu within the (+) and (-) growing chain were also considered. Illustrations of six possible conformations of the *i*Bu growing chain for the π-complex of 1,2-*re* and 1,2-*si* insertions in the presence of the salicylate donor (SID-4) are given in Figure 3. The Ti atom has the +3 oxidation state and contains one unpaired electron. Hence, the doublet spin state was set. Spin-unrestricted calculations for the open-shell systems were performed throughout this investigation using the same level of theory as described in Section 2.1. Also, the four MgCl_2_ units at the top layer, which involved donor and Ti coordination, were relaxed. The remaining MgCl_2_ units were frozen at the bulk MgCl_2_ value [54]. The flexible cluster model allows atoms to adjust their positions according to interactions with adsorbed molecules and it has been employed for the active surface of the Ziegler-Natta catalyst in several works [39,42,55]. The genuineness of the transition state was ensured by the presence of one imaginary frequency.

In this study, we focused only on the primary insertion modes, i.e., 1,2-*si* and 1,2-*re*, of propylene monomer into a Ti−*i*Bu. For both enantiofaces, the growing chain is oriented to minimize the steric interactions between the growing chain and the methyl group of the PP monomer. For example, in the case of 1,2-*re* enantioface, the methyl group of the PP monomer is on the opposite side to the growing chain and both methyl on the *i*Bu group avoid the methyl on the PP monomer which gives the least steric repulsion (see Figure 3, 1,2-*re*, A). Therefore, we will use the most stable structure of *i*Bu growing chain conformation with 1,2-*si* and 1,2-*re* of propylene insertions for investigation stereoselectivities and calculated activation energies of five salicylate donors.

## 3. Results and Discussion

### 3.1. Preferred Adsorption Modes of Salicylate Donors

Adsorption energies between isobutyl 2-benzyloxy-3,5-isopropyl benzoate or (SID-4) and the model of the pre-activated MgCl_2_ (110) surface for four adsorption modes, mono, chelate, bridge, and zip were calculated. Their values in kcal/mol together with O-Mg distances in Å were listed in Table 2. The SID-4 was selected to represent the salicylate donor since this compound gave the highest activity in the experiment, see Table 1 [16].

Adsorption energies of the mono, the chelate, the bridge, and the zip coordination modes of the SID-4 adsorbed to the pre-activated MgCl_2_ (110) surface are −30.2, −51.2 −45.9, and −42.0 kcal/mol, respectively. Thus, the most preferred adsorption mode for salicylate donors is the chelate mode, and the mono mode is the least favorite. The preferential binding mode of the salicylate donor to the MgCl_2_ (110) surface can be explained. The salicylate donor has two carbonyl oxygen atoms and large substituents. In the mono mode, the electron donor binds to the surface in the nearest vicinity of the titanium atom. Due to the steric repulsion, the mono mode forms the least stable complex, while the two carbonyl oxygens allow the electron donor to form a four-coordination complex with MgCl_2_ (110) surface that enhances the stability of the chelate mode. Unlike the case of monoester-type electron donors, such as methyl or ethyl benzoate, which have smaller substituents and one carbonyl oxygen, the mono coordination is the most preferred mode [14,26,42]. For the zip coordination, O_1_/O_2_ to Mg distances are longer than 4 Å. This suggests that the salicylate donor does not bind to the MgCl_2_ (110) surface. Nevertheless, the salicylate donor used one carbonyl oxygen at the O_3_ position bind to the Mg atom of the neighboring adsorption site on the MgCl_2_ (110) surface to reduce the steric interaction, caused by the large substituents of the donor. For the mono, chelate, and bridge coordination modes, distances between oxygen and Mg atoms are 1.99–2.09 Å for the carbonyl O_1_/O_3_ and 3.24–4.41 Å for the ether O_2_/O_4_. For all modes, the average carbonyl O-Mg distance is around 2.0 Å. This is understandable, since not only does the ether oxygen have less electron density (see O_1_–O_4_ charges in Table S1 in the supporting information), but there is also a strong repulsion between the ether moiety and the surface. However, it should be noted that the adsorption energy of the salicylate donor is irrespective of the coordinated distance.

### 3.2. Adsorption Energies of Five Salicylate Donors

In the last section, we carried out calculations to investigate the preferred adsorption modes of the salicylate donor. The calculations suggested that the chelate mode is energetically favorable. Therefore, the chelate mode was used as the adsorption mode for calculations of the adsorption energies of five salicylate donors. Adsorption energies (E_ads_) in kcal/mol and O-Mg distances in Å of the five salicylate donors adsorbed on the pre-activated MgCl_2_ (110) surface in the chelate mode are given in Table 3.

**Figure 4:**
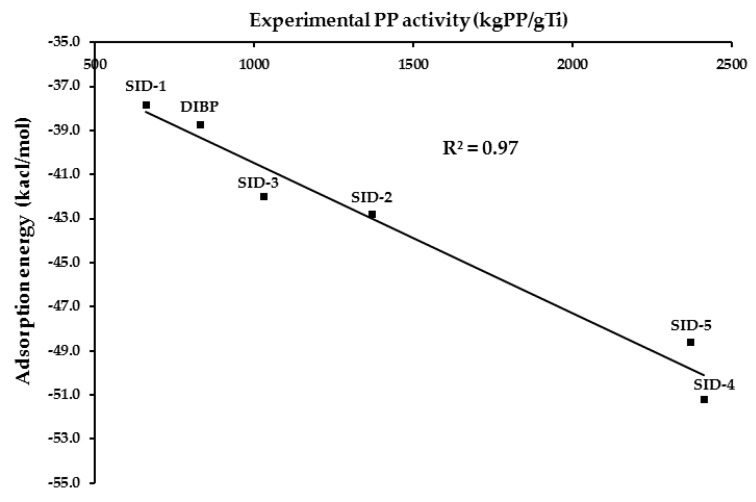
Relationship between the calculated adsorption energy and the experimental polypropylene (PP) activity of five salicylate (SID) and diisobutyl phthalate (DIBP) donors.

Lee et al. [20] found the relationship between adsorption energies of 1,3-diether donors to the MgCl_2_ surface and the isotacticity and the productivity of polypropylene. Moreover, Ratanasak et al. [56] have studied the relation between the adsorption energies of various classes of electron donors to the catalyst surface and activities. They found that the activity of polypropylene production linearly varies with the adsorption energy. We have made a plot between the experimental PP activity and the adsorption energy (E_ads_) of the five salicylate donors (SID-1–SID-5), together with that of diisobutyl phthalate (DIBP), as shown in Figure 4.

From Figure 4, the plot showed the squared correlation coefficient (R^2^) of 0.97. This high R^2^ value exhibits the linear relation between adsorption energies and PP activities. Hence, our hypothesis on the representative of the adsorption energy of the electron donor to the catalyst surface for the activity of the propylene polymerization is confirmed. Thus, the salicylate donor with the strongest adsorption energy (highest negative value) provides the highest activity for the ZN catalyst. This information is useful for the future design of new electron donors. Moreover, the adsorption energy of diisobutyl phthalate donor is just −38.7 kcal/mol) and three-fourths of our best salicylate donor (SID-4). Thus, DIBP is less active than most of the salicylate donors studied here. We analyzed the dependence of the adsorption energy with the substituent group at R_1_, R_2_, and R_3_ positions for five salicylate donors. The increase of the adsorption energy with the size of R_1_ and R_2_ substituents was observed. Having the same substituents at R_2_ and R_3_, while increasing the size of the substituent at R_1_ position from H (hydrogen) to Me (methyl), SID-3 has improved E_ads_ of SID-1 by 4.2 kcal/mol. Changing both H at R_1_ and R_2_ by *i*Pr, (isopropyl) E_ads_ of SID-4 is lowered from SID-1 by 13.4 kcal/mol and when changing by *t*Bu (tertiarybutyl) the adsorption is stronger by 10.8 kcal/mol for SID-5. However, when substituting R_3_ of SID-3 by *t*Bu, E_ads_ of SID-2 is enhanced by only 0.8 kcal/mol. Thus, to enhance the catalytic activity of ZN R_3_ of the salicylate donor can be either Ph (phenyl) or *t*Bu while R_1_ and R_2_ should be a bulky group. Our results demonstrated that the size of substituents plays a key role in controlling the adsorption energy. The salicylate donor, which provides strong adsorption energy, should have a bulky group at the R_1_ and R_2_ positions, and a phenyl or tertiary butyl group at the R_3_ position.

### 3.3. Activation Energies and Stereoselectivities

From Figure 3, the most stable *i*Bu growing chain conformation is 1,2-*re*, A. Thus, the 1,2-*re* propylene insertion on the front side is the preferred pathway for the study of the reactivity and stereoselectivity of five salicylate donors. Considering the 1,2-*si* insertion, the conformation 1,2-*si*, A is the most favorable route, but it has less stable energy than the 1,2-*re*, A conformation by 3.9 kcal/mol. For the π-complex, we observed that the position of isopropyl substituents on the donors is closer to the methyl moiety of PP in the *re* enantioface than in the *si* enantioface. Thus, the π-complex of the *re* enantioface is probably stabilized by the dispersion-type interaction between the methyl moiety and the substituents.

According to Cossee and Arlman [33,34,35] and Brookhart and Green [36], the insertion step involves the π-complex formation between the active catalyst and the olefin, the formation of the four-centered transition state, and the generation of the insertion product. Thus, the π-complex formation energy (ΔE_π_) and the intrinsic activation energy (E_a_) (the energy difference between the transition state structure (TS) and the π-complex) can be used to assess the reactivity of the ZN catalysts. Since the flexible cluster model is employed in this work, the π-complexation energy is then included the relaxation of the surface apart from the coordination energy. The stereoselectivity of polypropylene can be evaluated from the relative barrier (Rel.), which is the difference between the TS energy of 1,2-*si* (E_TS_(*si*)) and 1,2-*re* (E_TS_(*re*)) insertions. Additionally, it is also useful to estimate the apparent activation energy (E_a_(app)) which is ∆E_π_ + E_a_ and equivalent to the transition state energy relative to the dissociation channel of the catalyzed PP polymerization. Since the chelate mode was the preferred adsorption mode for salicylate donors, this mode was then selected for the construction of the active MgCl_2_ (110) surface. Table 4 lists π-complex formation energy, intrinsic activation energy, apparent activation energy, and relative barrier for the insertion step of the ZN-catalyzed PP polymerization with five salicylate donors.

**Figure 5:**
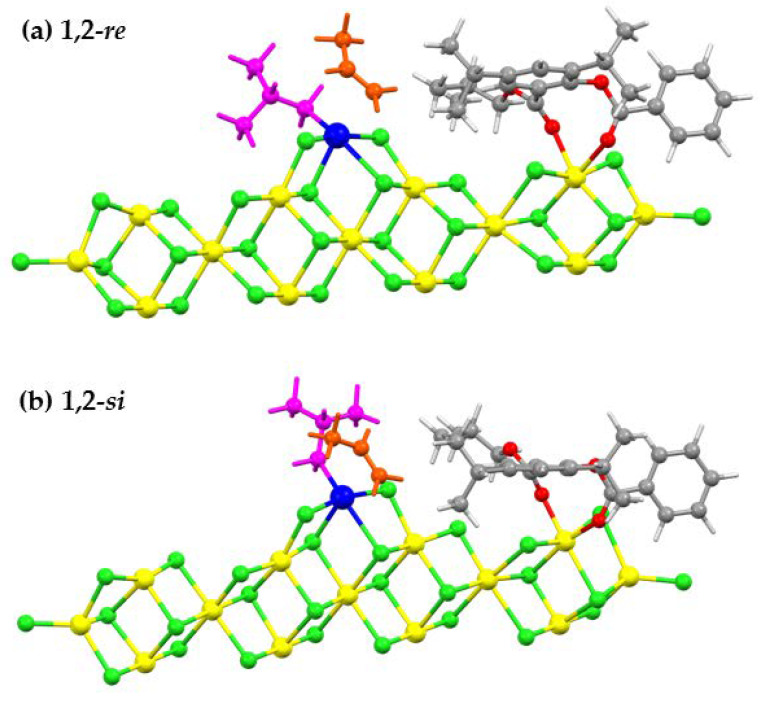
Structures of the π-complex of (**a**) 1,2-*re* and (**b**) 1,2-*si* insertions of the ZN-catalyzed PP polymerization with the salicylate (SID-4) donor.

The π-complex formation energies (ΔE_π_) of the five salicylate donors are in the range of −58–−64 kcal/mol. Generally, the 1,2-*re* insertion complex gives lower ΔE_π_ than its 1,2-*si* counterpart, suggesting the more stable π-complex of the 1,2-*re* insertion mode. Structures of the π-complex of (a) 1,2-*re* and (b) 1,2-*si* insertions of ZN-catalyzed PP polymerization with the salicylate (SID-4) donor are shown in Figure 5. From Figure 5, we observed that the position of isopropyl substituents on the donors is closer to the methyl moiety of PP in the *re* enantioface π-complex than in the *si* enantioface. Thus, the *re* enantioface π-complex is probably stabilized by the dispersion-type interaction between the methyl moiety and the substituents. The π-complex of 1,2-*re* insertion for the case of SID-3 provides the strongest ΔE_π_ (−63.5 kcal/mol), while that of 1,2-*si* for SID-2 gives the weakest interaction (−58.0 kcal/mol). There seems to be no direct relationship between activity and ΔE_π_. Comparing with DIBP (−37.4 kcal/mol for 1,2-*si* and −41.7 kcal/mol for 1,2-*re* insertions [23]), π-complex formation energies of all primary(1,2) insertion modes for five salicylates are larger. However, the π-complexation energy of the ZN without the electron donor is only −30 (*si* face) and −33 (*re* face) kcal/mol [17], which suggests that the electron donor stabilizes the π-complex.

Intrinsic activation energies (E_a_) of the five salicylate donors for both insertion modes are between 3.9 to 9.0 kcal/mol, and the 1,2-*re* insertion has a higher activation barrier than its corresponding 1,2-*si*. This similar trend was also found for the 1,2-*re* /1,2-*si* insertion with DIBP (4.7/4.5 kcal/mol) and dibenzoyl sulfide (6.5/4.4 kcal/mol) [23]. When we considered the relationship between the intrinsic activation energies of 1,2-*re* and 1,2-*si* insertions and the log of experimental PP activities of five salicylate donors, we found R^2^ = 0.98 and 0.97, respectively. Thus, the ln(activity) is directly related to the intrinsic activation barrier of the insertion step. (For the first-order kinetics, lnk = −E_a_/RT). The lowering of the activation barrier will enhance the activity of the ZN catalyst. There seems to be a relation between E_a_(app) and the ln(activity). We obtained R^2^ of 0.99 and 0.64 for the E_a_(app) of the 1,2-*re* and -*si* insertions, respectively. The activity is related to the TS energy of the 1,2-*re* insertion. Thus, the donor which can better stabilize TS energy of 1,2-*re* insertion will provide a higher activity for the catalyst. Interestingly, while ΔE_π_ does not have any relation to the activity, the E_a_ and E_a_(app) do. We also found a correlation between E_a_/E_a_(app) and E_ads_ to be around 0.94. This suggests that factors which provide high adsorption energy will be the same for E_a_/E_a_(app).

Moreover, we found the highest occupied molecular orbital (HOMO) energies of SID-1 to SID-5 donors (−0.249, −0.242, −0.247, −0.238 and −0.235 a.u., respectively) to be also linearly related to ln(PP activity) with R^2^ = 0.94. The donor with higher HOMO provides higher activity for the ZN catalyst. More interestingly, with the least square fit for E_a_ of 1,2-*re* and 1,2-*si* insertions and HOMO we obtained R^2^ of 0.94 and 0.87, respectively. Thus, HOMO data is better correlated with E_a_ of 1,2-*re* insertion. The relation between HOMO and E_a_ could be well explained by the Frontier Molecular Orbitals (FMO). This information suggests that the more potent salicylate donors should have high HOMO energy (less negative value). However, we should restrict our observation to within the same class of compounds.

**Figure 6:**
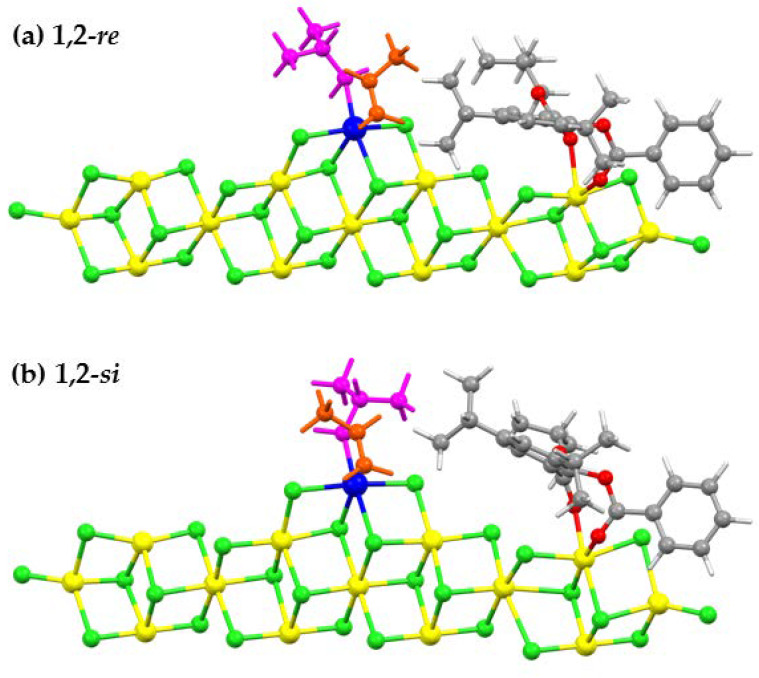
Structures of the transition sates of (**a**) 1,2-*re* and (**b**) 1,2-*si* insertions of ZN-catalyzed PP polymerization with salicylate (SID-4) donor.

Figure 6 illustrates the transition state structures of two enantiofaces of the primary (1,2) insertion modes of the ZN-catalyzed PP polymerization with SID-4. From the Figure, it appears that TS structures are similar to the corresponding π-complex structures, where the methyl moiety of PP is positioned furthest from the donor in the primary (1,2)-*si*, and in the primary (1,2)-*re* they are in the close vicinity. If the E_a_ for the insertion step is controlled solely by the steric interaction between the methyl moiety of PP and the substituents of the donor, as suggested by Cavallo et al. [39], the TS of the 1,2-*re* insertion should be less stable (higher) than that of the 1,2-*si*. From the values of E_a_(app) in Table 4, the energy of the TS of the 1,2-*re* insertion is lower than that of 1,2-*si* for all electron donors. In other words, the TS of the 1,2-*re* with salicylate donor is more stable than the TS of the 1,2-*si.* Thus, the close encounter of the methyl moiety of PP and substituents on the donor provides a favorable interaction to the TS of 1,2-*re* insertion. Thus, the dispersion-type interaction should be another effect. To prove our hypothesis, the π-complex formation energy (ΔE_π_), the intrinsic activation energy (E_a_), the relative barrier (Rel.), and the apparent activation energy (E_a_(app)) of the five salicylate donors (SID-1–5) were calculated using the B3LYP method. These values are given in Table S3 in supporting information. The values in Table S3 differ from those in Table 4. This suggests the importance of dispersion interaction. However, without dispersion (values in Table S3) the (1,2)-*re* remains the preferred insertion mode for salicylate donors (noticing from E_a_(app), and Rel.), in exception of SID-1. Thus, we still believed that the stereoselectivity of the ZN catalyst is controlled by the steric interaction between the electron donor and the methyl moiety of propylene, in agreement with Corradini [52], Cavallo [39], and the Taniike groups [26]. However, the (1,2)-*re* insertion gains extra stability from the dispersion interaction. Unlike when the B3LYP-D3 method was employed for the calculations, no relation between computed values of ΔE_π_ or E_a_ or Rel. or E_a_(app)) and the activities of the five salicylate donors was observed when the calculations were performed using B3LYP (see Table S4). This implies the significance of the dispersion-type interaction for this system. Therefore, we can conclude that there exists a dispersion-type interaction between the methyl moiety of PP and the substituents of the donors, which helps to stabilize the TS of the 1,2-*re* insertion. The higher activation energy of the 1,2-*re* insertion mainly comes from the stronger π-complex formation of the *re* enantioface. 

The polymerization can be determined by relative barriers (Rel.). Theoretically, higher Rel. would relate to the higher stereoselectivity. The relative barrier of ZN-catalyzed PP polymerization with the five salicylate donors of SID-1 to SID-5 are given in Table 4. Experimentally, the stereoselectivity can be indicated by the percent isotactic sequence length (%mm) and isotacticity index (%I.I). These values for the five salicylate donors are listed in Table 1. The positive value of the relative barrier indicates that the TS with a *re*-coordinated propylene face is more stable than the corresponding *si*-enantioface. From the result, the SID-4 system gave the highest value for the relative barrier (3.9 kcal/mol) and hence the highest stereoselectivity (%mm = 91.0 and %I.I = 98.6). The SID-1 system has the lowest relative barrier (1.1 kcal/mol) and the lowest selectivity (%mm = 85.5 and %I.I = 96.3). Compared with the industrial diisobutyl phthalate donor, we have previously reported that the transition state for the primary (1,2)-*re* is 4.0 kcal/mol below that for primary (1,2)-*si*. [23]. The %mm and %I.I of PP prepared by the diisobutyl phthalate internal donor are 91.0% and 91.7%, respectively [16]. Thus, the relative barrier of the ZN-catalyzed PP polymerization correlates well with the experimental stereospecificity data [16]. Also, the %selectivity, which should be closely related to both %mm and %I.I., can be estimated using the Curtin-Hammett principle (see the details in Table S6 of Supporting Information). The linear relationship between the %selectivity for the five salicylate donors and %mm and %I.I was observed with R^2^ of 0.74 and 0.55, respectively. Since the substituents on the electron donor play roles in the stability of the TS of the 1,2-*re* insertion as mentioned earlier, types of substituents on the donor can be used to assess the selectivity of the ZN catalyst. The SID-1 donor which has the lowest Rel. (1.0 kcal/mol) contains an H atom as the substituent for R_1_ and R_2_ and phenyl for R_3_. The SID-4 donor with *i*Pr on R_1_ and R_2_ while having the same substituent on R_3_ as the SID-1 gives the largest Rel. (3.9 kcal/mol). Similarly, with *t*Bu on R_2_ and R_3_, the SID-5 also has large Rel. (3.6 kcal/mol). Thus, the substituents on R_1_ and R_2_ enhance the stability of the TS for 1,2-*re* insertion towards that for 1,2-*si*. The *i*Pr substituent provides a more favorable interaction than *t*Bu. Possibly the *t*Bu is too bulky. The R_3_ substituent plays a smaller role than those on R_1_ and R_2_, since it gives a small difference for the relative barriers of SID-2 (3.5 kcal/mol) and SID-3 (3.2 kcal/mol). It should be noted that suggestions for improving stereoselectivity by salicylate electron donors are the same as those for improving activity. Therefore, the salicylate donor that provides high activity will also provide high stereoselectivity.

### 3.4. Comparing with Other Internal Electron Donors

The isobutyl 2-benzyloxy-3,5-isopropyl benzoate or salicylate donor (SID-4) was selected to represent five salicylate donors, since this compound gives the highest activity [16]. Apparent activation energies (E_a_(app)) of primary (1,2)-*re* insertion in kcal/mol, relative barrier (Rel.) in kcal/mol, activity in kg-PP/gCat for di-*n*-butyl-2-cyclopentyl malonate, dibenzoyl sulfide, diisobutyl phthalate donors and in kg-PP/gTi for diisobutyl phthalate and salicylate donors, and %isotacticity in %mmmm (pentrad) for di-*n*-butyl-2-cyclopentyl malonate, dibenzoyl sulfide, diisobutyl phthalate donors, and in %mm (triad) for diisobutyl phthalate and salicylate donors are listed in Table 5. In our previous work, we have shown that E_a_(app) can be used on par with the experimental activity [23]. Thus, it was used for the comparison of the activity of the ZN catalyst with various electron donors, whereas Rel. was utilized for comparing %isotacticity.

Among all internal electron donors in Table 5, the ZN catalyst with salicylate donor (SID-4) gives the lowest (highest negative) E_a_(app) and hence, the best activity. This is followed by those with dibenzoyl sulfide, di-*n*-butyl-2-cyclopentyl malonate, and diisobutyl phthalate donors, which have a higher E_a_(app) (less negative) and, thus, less activity. The apparent activation energy of the ZN catalyst without a donor is the least negative and, therefore, the catalyst shows the poorest activity. In the order of experimental activity, the list of donors is sulfide > malonate, phthalate. This order agrees with the calculated apparent activation energies. Unfortunately, the salicylate donor (SID-4) does not share the same unit for activity. Thus, it cannot be directly compared with other internal donors. By comparing E_a_(app), the ZN catalyst with the salicylate donor probably yields similar activity to that of sulfide donor. Using data of malonate, sulfide, and phthalate donors, we obtained a linear equation between ln(activity) and E_a_(app) with R^2^ of 0.98. From the equation, we estimated the activity of the ZN catalyst with salicylate donor to be 40.4 kg-PP/gCat.

Excluding data of the malonate donor in Table 5, it can be seen that the %Isotacticity is well predicted by the relative barrier (Rel.) [16]. The ZN with sulfide donor gives Rel. of 11.9 kcal/mol and has a %mmmm of 91.7, while that with phthalate donor gives Rel. of 4.0 kcal/mol and have %mmmm of 88.7 [23]. The salicylate donor which has comparable Rel. to the phthalate donor provides the polypropylene product with similar %isotacticity. Interestingly, the malonate donor has a negative relative barrier, implying that the TS for the 1,2-*si* insertion is more stable. Moreover, the ZN catalyst with the malonate donor yields the polypropylene product with better %isotacticity (%mmmm = 97.5) than that with the sulfide donor [13]. Thus, the interaction involved in the insertion step of the ZN-catalyzed PP polymerization with the malonate donor must be different from others.

The relative barrier can be decomposed to contributions from ΔE_π_ and E_a_. Since the transition state energy relative to the dissociation channel (E_TS_) is E_a_(app) and Rel. = E_TS_(*si*) − E_TS_(*re*). Therefore,

Rel. = Δ∆E_π_ + ∆E_a_

where ∆∆E_π_ = ∆E_π_(*si)* − ∆E_π_(*re*), and ∆E_a_ = E_a_(*si*) − E_a_(*re*). The decomposition of the relative barrier of malonate, sulfide, phthalate, and salicylate (SID-4) donors is given in Table 6.

The positive sign of the value means that the value for the *re* face of the propylene monomer is lower than that of the *si* face and vice versa. From Table 6, the contribution of ΔE_π_ to the relative barrier favors the *re* face insertion, while that of E_a_ favors the *si* face insertion for all donors. Thus, the nature of interactions that controlled the stereoselectivity is the same for all donors. However, for salicylate, sulfide, and phthalate donors, the magnitude of the ΔE_π_ contribution is larger than that of the E_a_ contribution. Thus, the selectivity is dictated by the contribution of ΔE_π_ and *re* face insertion is preferred. Whereas it is contrary to the malonate donor. Therefore, the selectivity is controlled by the E_a_ contribution and the *si* face insertion is preferred.

## 4. Conclusions

The preferred adsorption mode of salicylate donors is the chelate mode, similar to malonate [17], dibenzoyl sulfide [23], and diisobutyl phthalate [23] donors. The carbonyl O-Mg coordinated distances for the chelate mode are between 2.02–2.10 Å. A good linear relation between adsorption energies of the five salicylate donors and the experimental PP activity was noticed with R^2^ = 0.96, implying that salicylate donors with the strongest adsorption energy will provide the ZN catalyst with the highest activity. This finding is in line with our previous work on other donors [23]. Salicylate donors (SID-2 to SID-5) have lower adsorption energies (−42.8 to −51.2 kcal/mol) than that of the diisobutyl phthalate (DIBP) donor (−38.7 kcal/mol). Our results also demonstrated that the size of substituents plays a key role in controlling the adsorption energy. The salicylate donor which provides strong adsorption energy should have a bulky group at the R_1_ and R_2_ positions and a phenyl or tertiary butyl group at the R_3_ position.

The insertion step which is the RDS for olefin polymerization involves the π-complex formation and the activation of olefin insertion. Thus, the activity of PP polymerization can be estimated from the π-complex formation energy (∆E_π_) and the intrinsic activation energy (E_a_). The ∆E_π_ for the five salicylate donors were reported to be between −58.0–−59.5 kcal/mol and −62.6–−63.5 kcal/mol for the *si* and the *re* enantioface complexes, respectively. Most salicylate donors have a stronger π-complexation energy than DIBP. The intrinsic activation energy for 1,2-*si* and -*re* insertions of the ZN with the five salicylate donors are 3.9–6.4 and 4.6–9.0 kcal/mol, respectively. The corresponding apparent activation energies in the respective order of 1,2-*si* and 1,2-*re* insertions are −53.1–−55.3 and −54.1–−58.6 kcal/mol. No relation between ∆E_π_ and activity was observed. However, we found the linear relation between the intrinsic activation energy and the ln(experimental activity) with R^2^ of 0.98 and 0.97 for the primary(1,2)-*re* and (1,2)-*si* insertion, respectively. The apparent activation energy (E_a_(app)) which is ∆E_π_ + E_a_ and equivalent to the transition state (TS) energy in relative to the dissociation channel of five donors has the R^2^ with the ln(activity) of 0.99 and 0.64 for the 1,2-*re* and (1,2)-*si*, respectively. Thus, the activity also strongly correlates with E_a_(app) for 1,2-*re* insertion. For E_a_ and E_a_(app), the lowering of the value causes an increase in the activity of the ZN catalyst. The highest occupied molecular orbital (HOMO) energy of the salicylate donor was as well found to show the strong correlation with both the ln(activity) (R^2^ = 0.94) and E_a_ of 1,2-*re* insertion (R^2^ = 0.94). The relation between HOMO and E_a_ could be explained by the frontier molecular orbital. Moreover, the relation between E_a_/E_a_(app) and adsorption energies was observed (R^2^ = 0.94). Thus, electron donors which have strong adsorption to the catalyst also provide low E_a_/E_a_(app) for the insertion step of PP polymerization.

The stereoselectivity can be estimated from the relative stability between TS structures of 1,2-*si* and -*re* insertions or the relative barrier (Rel.). For the five salicylate donors, the transition state of the 1,2-*re* insertion has lower energy than that of the 1,2-*si* insertion. Thus, the salicylate donors provide stereoselectivity for the ZN catalyst. Relative barriers of the five salicylate donors are 1.0 (SID-1) to 3.9 (SID-4) kcal/mol. Thus, SID-4 provides the best stereoselectivity for this series. Furthermore, Rel. can be used to predict %Isotacticity. It provides R^2^ of 0.79 and 0.61 with the percent isotactic sequence length (%mm) and isotacticity index (%I.I), respectively. Relative barriers for SID-4 and DIBP are 3.9 and 4.0 kcal/mol, respectively, is following the experimental %mm of 91.0 and 91.7%. Therefore, the salicylate donor with the largest relative barrier will give the highest stereoselectivity. We found similar substituent effects in controlling activity and stereoselectivity. The electron donor which can yield high productivity will also give high stereoselectivity.

The apparent activation energy and the relative barrier can also be used to predict the activity and stereoselectivity of other electron donors. The order of donors according to the activity is isobutyl 2-benzyloxy-3,5-isopropyl benzoate (SID-4) > dibenzoyl sulfide (sulfide) > di-*n*-butyl-2-cyclopentyl malonate (malonate) > diisobutyl phthalate (phthalate). For the selectivity (excluding malonate), the order is sulfide > phthalate > salicylate. The relative barrier can be decomposed to contributions from τηε π-complex formation (∆∆E_π_) and insertion activation energy (∆E_a_). The ∆∆E_π_ prefers *re* enantioface while the ∆E_a_ prefers *si* enantioface. For most donors, the magnitude of ∆∆E_π_ is larger than ∆E_a_. Therefore, 1,2-*re* insertion is favored. For the malonate donor, ∆E_a_ is larger and the 1,2-*si* insertion is preferred.

## Figures and Tables

**Table 1:** Salicylate donors (SID) with different substituent groups at R_1_, R_2_ and R_3_ positions and experimental data [16].

SID	R_1_	R_2_	R_3_	Activity (kgPP gTi^−1^)	%mm ^a^	%I.I. ^b^
**SID-1**	H	H	Ph	660	85.5	96.3
**SID-2**	Me	H	*t*Bu	1370	88.1	96.9
**SID-3**	Me	H	Ph	1030	89.6	98.0
**SID-4**	*i*Pr	*i*Pr	Ph	2410	91.0	98.6
**SID-5**	*t*Bu	*t*Bu	Ph	2370	88.9	97.7

^a^ %mm denotes the average meso (isotactic) sequence length or % triads of polypropylene from ^13^C-NMR [16]. ^b^ %I.I. denotes % of the isotactic index was tested by extraction with boiling *n*-heptane for 6 h [16].

**Table 2:** Adsorption energies (E_ads_) and O-Mg distances of salicylate donor (SID-4) adsorbed on the pre-activated MgCl_2_ (110) surface with four different adsorption modes.

Adsorption Mode	E_ads_ (kcal/mol)	Distance (Å)			
		O_1_-Mg	O_2_-Mg	O_3_-Mg	O_4_-Mg
Mono	−30.2	1.99	4.04	4.49	4.41
Chelate	−51.2	2.03	4.17	2.09	3.58
Bridge	−45.9	2.03	3.74	2.06	3.24
Zip	−42.0	5.58	4.06	1.98	4.17

**Table 3:** Adsorption energies (E_ads_) and O-Mg distances of the five salicylate donors (SID-1–SID-5) and diisobutyl phthalate (DIBP) adsorbed on the pre-activated MgCl_2_ (110) surface with the preferred chelate mode.

Chelate Mode	E_ads_ (kcal/mol)	Distance (Å)			
		O_1_-Mg	O_2_-Mg	O_3_-Mg	O_4_-Mg
SID-1	−37.8	2.04	4.18	2.08	3.64
SID-2	−42.8	2.05	4.18	2.10	3.61
SID-3	−42.0	2.04	4.18	2.09	3.58
SID-4	−51.2	2.03	4.17	2.09	3.58
SID-5	−48.6	2.02	4.17	2.09	3.67
DIBP	−38.7	2.03	3.78	2.07	4.01

**Table 4:** The π-complex formation energy (ΔE_π_), the intrinsic activation energy (E_a_), the relative barrier (Rel.), and the apparent activation energy (E_a_(app)) of the ZN-catalyzed PP polymerization with the five salicylate donors together with values of transition state imaginary frequencies (*ν*).

SID	Insertion	ΔE_π_ (kcal/mol)	E_a_ (kcal/mol)	E_a_(app) (kcal/mol)	Rel. (kcal/mol)	*ν* (cm^−1^)
**1**	1,2-*si*	−59.5	6.4	−53.1	1.1	−340*i*
	1,2-*re*	−63.1	9.0	−54.1		−339*i*
**2**	1,2-*si*	−58.0	5.1	−52.9	3.5	−344*i*
	1,2-*re*	−62.6	6.1	−56.5		−354*i*
**3**	1,2-*si*	−58.1	5.7	−52.4	3.2	−331*i*
	1,2-*re*	−63.5	7.9	−55.6		−345*i*
**4**	1,2-*si*	−59.2	3.9	−55.3	3.9	−332*i*
	1,2-*re*	−63.2	4.6	−58.6		−340*i*
**5**	1,2-*si*	−58.8	4.4	−54.5	3.6	−362*i*
	1,2-*re*	−62.7	4.8	−57.9		−375*i*

**Table 5:** Apparent activation energy (E_a_(app)) of primary (1,2)*-re* insertion, relative barrier (Rel.), experimental activity, and %isotacticity of ZN-catalyzed PP polymerization in absence of donor (*w*/*o* donor) and with malonate, sulfide, phthalate, and salicylate donors.

1,2-*re* Insertion	Theoretical Predictions		Ref.	Experiments		Ref.
	E_a_(app) (kcal/mol)	Rel. (kcal/mol)		Activity (kg-PP/gCat)	%Isotacticity (%mmmm)	
*w*/*o* donor	−26.6	0.2	[17]	-	-	
Malonate ^i^	−38.5 ^ac^	−1.5	[17]	25	97.5	[13]
Sulfide ^ii^	−58.2	11.9	[23]	40	91.7	[57]
Phthalate ^iii^	−37.0	4.0	[23]	22	88.7	[57]
				830 ^b^	91.7 ^c^	[16]
Salicylate ^iv^	−58.6	3.9		2410 ^b^	91.0 ^c^	[16]

^i^ Di-*n*-butyl-2-cyclopentyl malonate; ^ii^ Dibenzoyl sulfide; ^iii^ Diisobutyl phthalate; ^iv^ Isobutyl 2-benzyloxy-3,5-isopropyl benzoate (SID-4); ^a^ primary(1,2)-*si* insertion; ^b^ Activity in (kg-PP/gTi); ^c^ %Isotacticity in %mm.

**Table 6:** Differences between *re* and *si* enantiofaces of π-complexation energies (ΔΔE_π_), intrinsic activation energies (ΔE_a_), and transition state energies of the primary (1,2) insertion step (relative barrier, Rel.).

Electron Donor	ΔΔE_π_ (kcal/mol)	ΔE_a_ (kcal/mol)	Rel. (kcal/mol)
Malonate	1.6	−3.1	−1.5
Sulfide	14.0	−2.1	11.9
Phthalate	4.3	−0.3	4.0
Salicylate	3.9	−0.7	3.9

## Supplementary Materials

The following are available online at https://www.mdpi.com/2073-4360/12/4/883/s1, Figure S1: Chelate adsorption modes of five salicylate donors (SID-1−SID-5) and diisobutyl phthalate (DIBP) donor on the ZN catalyst, Table S1: NBO charges on the O_1_-O_4_ oxygen of five salicylate donors (SID-1−SID-5) and diisobutyl phthalate (DIBP) donor, Table S2: Adsorption energies (E_ads_) of five salicylate donors (SID) with different substituent groups at R_1_, R_2_ and R_3_ positions and experimental data [16] adsorbed on the pre-activated MgCl_2_(110) surface with the preferred chelate mode using B3LYP-D3 and B3LYP, Table S3: The π-complex formation energy (ΔE_π_), the intrinsic activation energy (E_a_), the relative barrier (Rel.), and the apparent activation energy (E_a_(app)) of the ZN catalyzed PP polymerization with five salicylate donors together using B3LYP calculations, Table S4: The correlation between calculated parameters (E_ads_, E_a_, ΔE_π_ E_a(app)_, and Rel.) using the B3LYP-D3 calculations and experimental results (Activity, %mm and %I.I.) of five salicylate donors (SID) from reference [16], Table S5: The correlation between calculated parameters (E_ads_, E_a_, ΔE_π_ E_a(app)_, and Rel.) using the B3LYP calculations and experimental results (Activity, %mm and %I.I.) of five salicylate donors (SID) from reference [16]., Table S6: Five salicylate donors (SID) and %mm and %I.I. from the experimental results [16] and the relative barrier (Rel.) from B3LYP-D3 calculations and % selectivity from eq. 1 at temperature 343 K.
