# Supplementary material for: Roles of Salicylate Donors in Enhancement of Productivity and Isotacticity of Ziegler–Natta Catalyzed Propylene Polymerization
Source: Polymers (Basel). 2020 Apr 10;12(4):883. doi: 10.3390/polym12040883 (PMC7240445; doi:10.3390/polym12040883)
Supplement: Supplementary file 1 [file polymers-12-00883-s001.pdf]

Supporting Information

Roles of Salicylate Donors in Enhancement of Productivity and Isotacticity of Ziegler–Natta Catalyzed Propylene Polymerization

Manussada Ratanasak [1], Jun-ya Hasegawa [1] and Vudhichai Parasuk [2,*]

[1] Institute for Catalysis, Hokkaido University, Kita 21, Nishi 10, Sapporo, Hokkaido 001-0021, Japan; manussada@cat.hokudai.ac.jp (M.R.); hasegawa@cat.hokudai.ac.jp (J.H.)

[2] Center of Excellence in Computational Chemistry, Department of Chemistry, Faculty of Science, Chulalongkorn University, Bangkok 10330, Thailand

* Correspondence: vudhichai.p@chula.ac.th; Tel.: +66-2218-7603

Table S1. NBO charges on the O_1-O_4 oxygen of five salicylate donors (SID-1–SID-5) and diisobutyl phthalate (DIBP) donor.

	NBO charge on			
**Donor**	**O_1**	**O_2**	**O_3**	**O_4**
**SID-1**	-0.582	-0.557	-0.589	-0.541
**SID-2**	-0.579	-0.557	-0.604	-0.553
**SID-3**	-0.598	-0.558	-0.585	-0.543
**SID-4**	-0.599	-0.558	-0.582	-0.548
**SID-5**	-0.602	-0.557	-0.581	-0.544
**DIBP**	-0.583	-0.553	-0.582	-0.559

Table S2. Adsorption energies (E_{ads}) of five salicylate donors (SID) with different substituent groups at R_1, R_2 and R_3 positions and experimental data [16] adsorbed on the pre-activated $MgCl_2$(110) surface with the preferred chelate mode using B3LYP-D3 and B3LYP.

**SID**	**R_1**	**R_2**	**R_3**	**Activity (kgPP gTi^{-1})**	**E_{ads} (kcal/mol)**	
					**B3LYP-D3**	**B3LYP**
SID-1	H	H	Ph	660	-46.4	**-33.9**
SID-2	Me	H	*t*Bu	1370	-50.5	**-39.2**
SID-3	Me	H	Ph	1030	-49.0	**-36.8**
SID-4	*i*Pr	*i*Pr	Ph	2410	-62.3	**-33.0**
SID-5	*t*Bu	*t*Bu	Ph	2370	-62.0	**-32.1**

Table S3. The π-complex formation energy (ΔE_π), the intrinsic activation energy (E_a), the relative barrier (Rel.), and the apparent activation energy (E_a(app)) of the ZN catalyzed PP polymerization with five salicylate donors together using B3LYP calculations.

**SID**	**insertion**	**ΔE_π (kcal/mol)**	**E_a (kcal/mol)**	**E_a(app) (kcal/mol)**	**Rel. (kcal/mol)**

**1**	1,2-*si*	-29.6	6.5	-23.2	-0.3
	1,2-*re*	-34.7	11.8	-22.8	
**2**	1,2-*si*	-27.5	6.4	-21.2	4.1
	1,2-*re*	-33.1	7.9	-25.3	
**3**	1,2-*si*	-29.4	6.5	-23.0	2.1
	1,2-*re*	-34.6	9.4	-25.1	
**4**	1,2-*si*	-38.0	6.6	-31.4	1.5
	1,2-*re*	-42.4	9.5	-32.9	
**5**	1,2-*si*	-35.6	5.9	-29.7	2.6
	1,2-*re*	-40.5	8.2	-32.3	

Table S4. The correlation between calculated parameters (E_{ads}, E_a, ΔE_{π} $E_{a(app)}$, and Rel.) using the B3LYP-D3 calculations and experimental results (Activity, %mm and %I.I.) of five salicylate donors (SID) from reference [16].

**Correlation between**		**R^2**	**Correlation between**		**R^2**
Activity	E_{ads}	0.96			
Activity	Ea of 1,2-*re*	0.94	Activity	Ea of 1,2-*si*	0.94
Activity	ΔE_{π} of 1,2-*re*	0.11	Activity	ΔE_{π} of 1,2-*si*	0.02
Activity	Ea(app) of 1,2-*re*	0.97	Activity	Ea(app) of 1,2-*si*	0.77
E_{ads}	Ea of 1,2-*re*	0.90	E_{ads}	Ea of 1,2-*si*	0.96
E_{ads}	Ea(app) of 1,2-*re*	0.97	E_{ads}	Ea(app) of 1,2-*si*	0.75
ln (Activity)	E_{ads}	0.95			
ln (Activity)	Ea of 1,2-*re*	0.98	ln (Activity)	Ea of 1,2-*si*	0.97
ln (Activity)	Ea(app) of 1,2-*re*	0.99	ln (Activity)	Ea(app) of 1,2-*si*	0.64
ln (Activity)	HOMO (SID1-5)	0.94			
Ea of 1,2-*re*	HOMO (SID1-5)	0.94	Ea of 1,2-^{si}	HOMO (SID1-5)	0.87
%mm	Rel.	0.79			
%I.I.	Rel.	0.61			

Table S5. The correlation between calculated parameters (E_{ads}, E_a, ΔE_{π} $E_{a(app)}$, and Rel.) using the B3LYP calculations and experimental results (Activity, %mm and %I.I.) of five salicylate donors (SID) from reference [16].

**Correlation between**		**R^2**	**Correlation between**		**R^2**
Activity	E_{ads}	0.27			
Activity	Ea of 1,2-*re*	0.32	Activity	Ea of 1,2-*si*	0.09
Activity	ΔE_{π} of 1,2-*re*	0.79	Activity	ΔE_{π} of 1,2-*si*	0.75
Activity	Ea(app) of 1,2-*re*	0.97	Activity	Ea(app) of 1,2-*si*	0.77
E_{ads}	Ea of 1,2-*re*	0.10	E_{ads}	Ea of 1,2-*si*	0.08
E_{ads}	Ea(app) of 1,2-*re*	0.38	E_{ads}	Ea(app) of 1,2-*si*	0.68
ln (Activity)	E_{ads}	0.14			
ln (Activity)	Ea of 1,2-*re*	0.47	ln (Activity)	Ea of 1,2-*si*	0.09
ln (Activity)	Ea(app) of 1,2-*re*	0.91	ln (Activity)	Ea(app) of 1,2-*si*	0.63
%mm	Rel.	0.16			
%I.I.	Rel.	0.04			

Curtin-Hammett principle: K= e$^{(-\Delta G)/RT}$

where ΔG = relative Gibbs energy, R = gas constant, and T = 343 K.

The %selectivity for A ⇌ B is computed from

$$\%\text{selectivity} = \frac{[A]}{[A]+[B]} x100 = \frac{1}{1+K} x100 \qquad \text{------------------ (1)}$$

Here, we did not determine Gibbs energy. Therefore, ΔG in the Curtin-Hammett principle is replaced by ΔE or Rel. (relative barrier). We considered the use of Rel. for ΔG is to the good approximation. Since S and ZPE correction would be similar for molecules with similar structure. Therefore, their contribution to ΔG would be minimal. The computed %selectivity were listed in Table S6. We have examined the correlation between % selectivity and %mm and %I.I. and R^2=0.74 and R^2=0.55, respectively, were resulted.

Table S6. Five salicylate donors (SID) and %mm and %I.I. from the experimental results [16] and the relative barrier (Rel.) from B3LYP-D3 calculations and % selectivity from eq. 1 at temperature 343 K.

SID	%mm	%I.I.	Rel. (kcal/mol)	%selectivity
**SID-1**	85.5	96.3	1.1	83.40
**SID-2**	88.1	96.9	3.5	99.41
**SID-3**	89.6	98.0	3.2	99.09
**SID-4**	91.0	98.6	3.9	99.67
**SID-5**	88.9	97.7	3.6	99.49

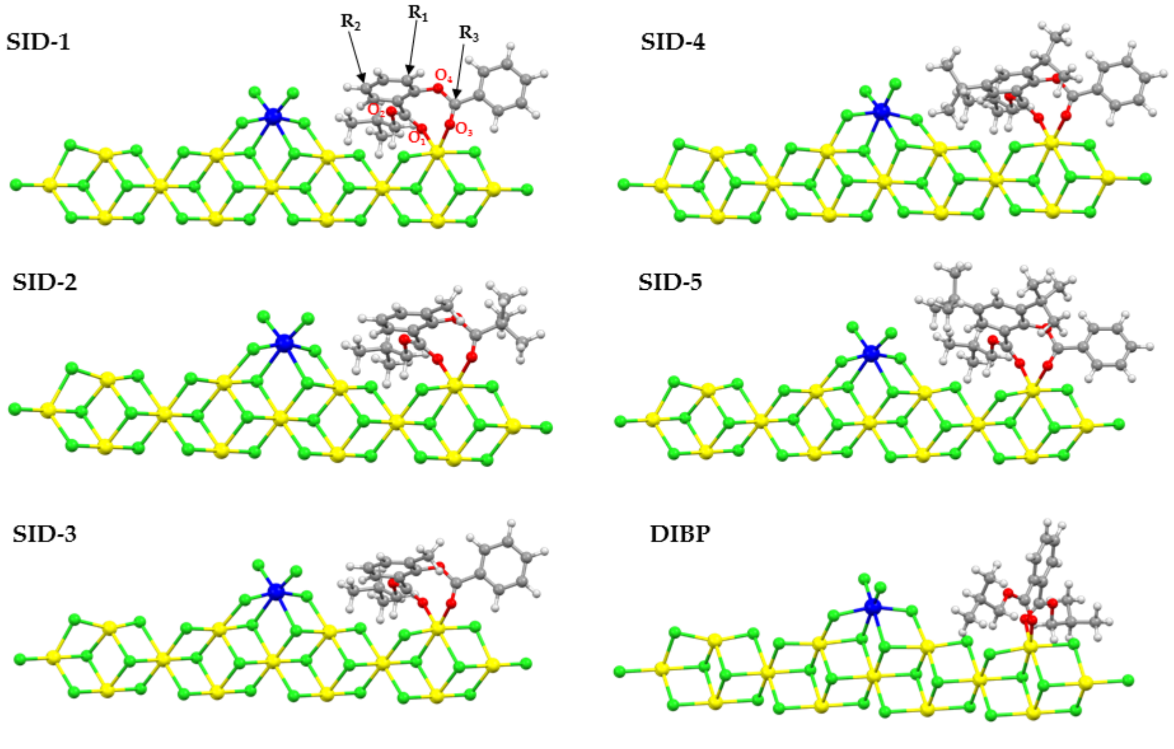

Figure S1. Chelate adsorption modes of five salicylate donors (SID-1–SID-5) and diisobutyl phthalate (DIBP) donor on the ZN catalyst. Color key: Mg, yellow; Ti, blue; Cl, green; O, red; C, gray; H, white.
